# Subacute Bacterial Cellulitis With a Subacute Clinical Course With Difficulty in Distinguishing From Sjögren’s Syndrome: A Case Report

**DOI:** 10.7759/cureus.33554

**Published:** 2023-01-09

**Authors:** Yuta Horinishi, Junko Yurizawa, Chiaki Sano, Ryuichi Ohta

**Affiliations:** 1 Community Care, Unnan City Hospital, Unnan, JPN; 2 Family Medicine, International University of Health and Welfare, Tokyo, JPN; 3 Community Medicine Management, Shimane University Faculty of Medicine, Izumo, JPN; 4 Communiy Care, Unnan City Hospital, Unnan, Shimane, JPN

**Keywords:** sjögren's syndrome, general medicine, rural hospital, subacute progressive course, staphylococcal cellulitis

## Abstract

Differentiating between infectious and autoimmune diseases regarding inflammatory changes in the soft tissues around the joints that develop in a subacute course is often difficult. Herein, we present the case of a 65-year-old woman who presented with a chief complaint of the tarsal metatarsal joint (Lisfranc joint) pain in the second, third, and fourth toes of her right foot that had persisted for several days. The patient was initially treated with non-steroidal anti-inflammatory drugs, colchicine, and prednisolone 10 mg for pseudogout and Sjogren's syndrome arthritis; however, there was little improvement. A few weeks later, the skin of the fingers and toes peeled, and the patient was diagnosed with subacute burn-like skin syndrome and treated with antibiotics. After the treatment, the inflammatory findings steadily improved. Arthritis associated with an infection is considered to have an acute course. However, subacute-to-chronic arthritis associated with Staphylococcus aureus infection may also be possible, as in this case. Therefore, the possibility of infection should be evaluated in patients with subacute-to-chronic refractory arthritis.

## Introduction

The subacute course of an infectious disease can be difficult to diagnose. Arthritis is classified as mono-, oligo-, or polyarthritis (with four or more joints), with an acute or chronic course having a six-week time window. Acute monoarthritis, bacterial infection, crystallization, traumatic injury, and early stages of chronic arthritis, such as collagen disease and malignancy, can be differentiated [1]. For example, Staphylococcus aureus is a major cause of bacteremia, infective endocarditis, bone, soft tissue, lung, and artifact infections with an acute course [2]. The likelihood of a bacterial infection decreases with time. In contrast, some infections that enter the epidermis and skin have subacute to chronic courses, such as infective endocarditis and osteomyelitis, which are caused by epidermal commensal bacteria [3].

Infectious diseases may have a subacute course, with the epidermis as the portal of access, and it is important to distinguish them from collagen diseases. As the direction of treatment is opposite for infectious and collagen diseases, appropriate diagnosis and treatment are important. Herein, we report the case of a 65-year-old woman with tarsal metatarsalgia of her right foot's second, third, and fourth toes that persisted for several days. The patient was diagnosed with staphylococcal cellulitis associated with Sjögren syndrome. In this case, the diagnosis was difficult because a subacute infection complicated arthritis. We report this case because we believe that the case history and method used to make the differential diagnosis could be useful in the future medical care of older adults.

## Case presentation

A 65-year-old woman presented to our hospital with the chief complaint of right ankle joint pain that had persisted for several days. When the patient visited our hospital, spontaneous pain and tenderness were evident in the right dorsal foot and metatarsophalangeal joints (Lisfranc joints) of her right foot's second, third, and fourth toes. Joint radiography revealed no fractures or erosion. Her laboratory data showed a high C-reactive protein and erythrocyte sedimentation rate. The patient was thus treated with non-steroidal anti-inflammatory drugs (NSAIDs), colchicine, and prednisolone (PSL) 10 mg for one week; however, her symptoms did not improve. Therefore, a detailed history and physical examination were performed again. A review of the system revealed the following: sunburn, skin rashes, occasional arthralgia for several years, excessive eye fat, and dental caries. Negative findings included Raynaud's loss, weight loss, and night sweats. Her medical history included hypertension, dyslipidemia, diabetes mellitus, fatty liver, hyperprolactinemia, Graves' disease after subtotal thyroidectomy (45 years prior), atrophic gastritis, and cataract. Medications included amlodipine besylate 5 mg, imidapril hydrochloride 5 mg, atorvastatin calcium hydrate 10 mg, linagliptin 5 mg, and levothyroxine sodium hydrate 25 μg.

Her vital signs were as follows: blood pressure, 156/69 mmHg; heart rate, 86 beats/min; respiratory rate, 16 breaths/min; body temperature, 36.8°C; and transcutaneous arterial blood oxygen saturation, SpO_2_ 98% (room air). Physical examination revealed a large amount of exudate and dryness of the oral cavity. No obvious abnormalities were observed in the chest or the abdomen. There was no sacroiliac joint tenderness or tenderness at the Achilles tendon attachment sites of the extremities. The left fingernail showed psoriatic changes, no nail epithelial capillary telangiectasia, and swelling of the metacarpophalangeal and distal interphalangeal joints in both fingers. The extensor joints showed Kevnell's sign and no ankle joint range of motion restrictions. Tenderness and a limited range of motion of the metatarsophalangeal joints of the right foot's second, third, and fourth toes were observed. The blood test results are presented in Table 1. An elevated inflammatory response and increase in anti-SS-A antibody levels were observed.

**Table 1:** Laboratory data of the patient

Parameters	Level	Reference
White blood cells	11.7	3.59.1 × 10^3^/μL
Neutrophils	66.8	44.0–72.0%
Lymphocytes	28.0	18.0–59.0%
Monocytes	5.1	0.0–12.0%
Eosinophils	0.0	0.0–10.0%
Basophils	0.1	0.0–3.0%
Red blood cells	3.81	3.76–5.50 × 10^6^/μL
Hemoglobin	12.8	11.3–15.2 g/dL
Hematocrit	38.6	33.4–44.9%
Platelets	34.4	13.0–36.9 × 10^4^/μL
Creatinine kinase	21	56–244 U/L
Antibody to Sjögren’s syndrome A	138	<10.0 U/mL
Antibody to Sjögren’s syndrome B	2.7	<10.0 U/mL
Complement 3	145	86-160 mg/dL
Complement 4	35	17-45 mg/mL
KL-6	272	105.3-401.2 U/mL
MPO-ANCA	<1.0	< 3.5 U/mL
Rheumatoid factor	0	< 15 IU/mL
Immunoglobin G	1983	870–1700 mg/dL
Immunoglobin E	5081	<170 U/mL
Immunoglobin A	448	110-410 mg/dL
Immunoglobin G4	68	5.3-116 mg/dL
Anti-citrullinated protein antibody	<0.6	< 5 U/mL

**Figure 1:**
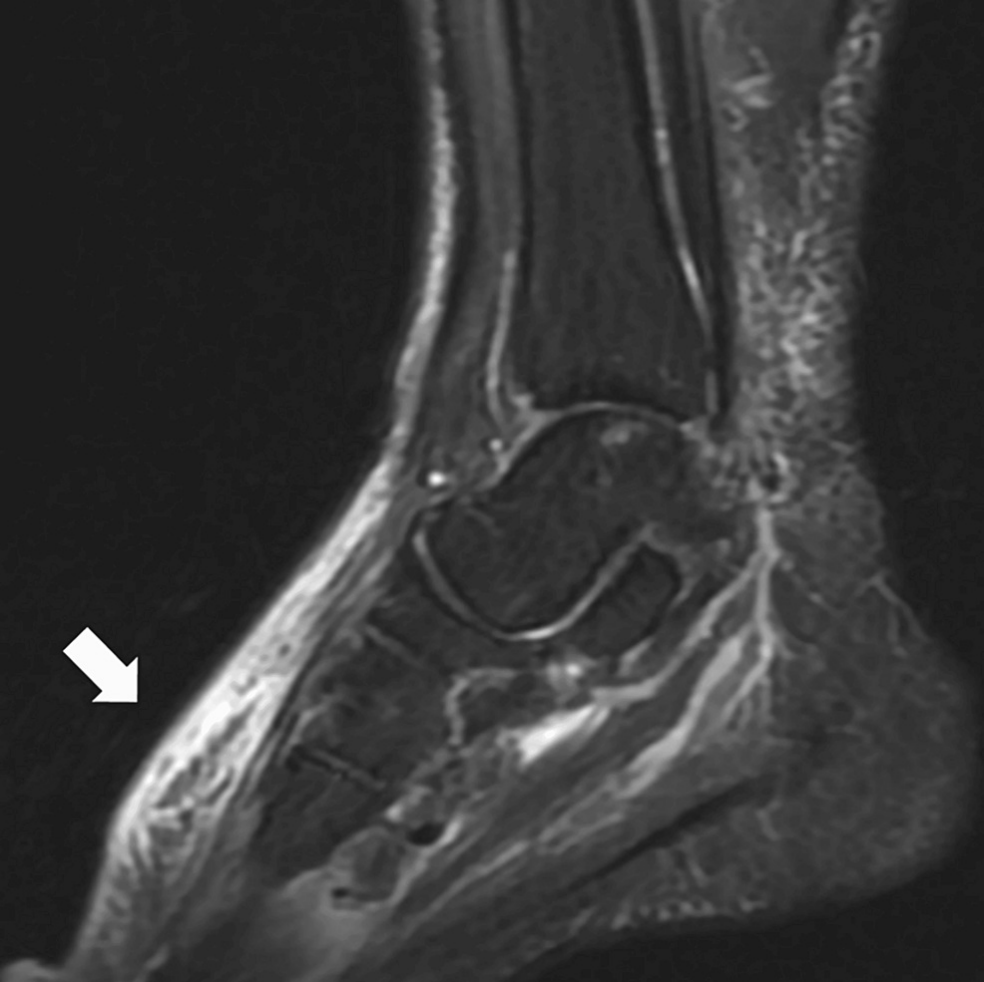
Right foot magnetic resonance imaging showing high signal range in the soft tissue of the right dorsal foot (white arrow)

On the second day of hospitalization, a salivary gland biopsy was performed based on the possibility of arthritis due to Sjögren's syndrome because of the presence of a large amount of eye exudate, dental caries, and positive anti-SS-A antibody. A minor salivary gland biopsy revealed an inflammatory infiltration of lymphocytes and plasma cells around the conduit, and Sjögren's syndrome was diagnosed. NSAIDs and PSL 10 mg were ineffective against arthritis. Pseudogout and psoriatic arthritis, rheumatoid factor, and anti-CCP antibodies were negative, and bone erosion was unremarkable. On the third day, a magnetic resonance imaging (MRI) scan of the ankle joint was performed, and the fat-suppressed images showed a high accumulation of soft tissue in the dorsum of the foot, in addition to Lisfranc's joint (Figure 1).

**Figure 2:**
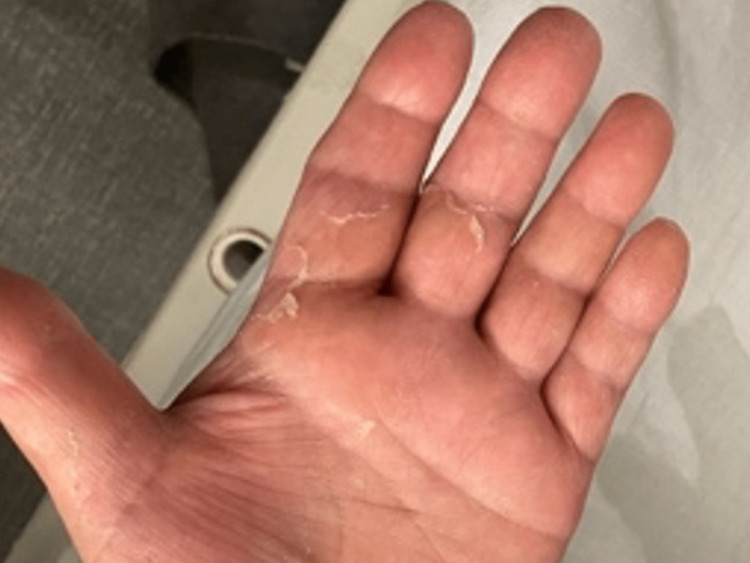
Exfoliation of the palm of the left hand showing burn-like skin syndrome

On the fourth day, the patient was diagnosed with burn-like skin syndrome with a subacute course due to the peeling of the skin on the fingers and toe tips (Figure 2).

Cefazolin 6 g/day was administered intravenously initially and shifted to oral cephalexin of 2g/day. The patient's heat sensation, swelling, and pain decreased the day after treatment. On the 10th day, the patient was discharged because her symptoms improved, and she could walk.

## Discussion

Staphylococcal and streptococcal infections of the skin and soft tissues generally worsen over hours to days, and systemic symptoms such as fever, tachypnea, hypotension, and tachycardia often occur [2]. The patient initially presented with acute onset monoarthritis and was treated for pseudogout; however, there was little improvement. There were no abnormalities in vital signs such as tachypnea or hypotension; however, after a few weeks, skin peeling occurred on the tips of the fingers. S. aureus produces epidermolytic toxin A (exfoliative toxin A) that acts on the systemic skin via the bloodstream and cleaves desmoglein 1, a desmosomal component protein. Further, it causes spicule melting in the upper epidermis, similar to pemphigus foliaceus, resulting in dermabrasion and intraepidermal blistering [2].

S. aureus is a major cause of bacteremia, infective endocarditis, bone, soft tissue, lung, and artifact infections with an acute course [2]. The likelihood of a bacterial infection is expected to decrease over time. However, in addition to acute S. aureus infections, chronic conditions also involve persistent cells. S. aureus adheres to host cell surfaces and subsequently forms biofilms via growth and matrix formation. The host immune system kills the bacteria outside the biofilm but cannot contact bacteria within the biofilm. Antibiotics penetrate the biofilms and kill most bacteria, but some remain unsterilized due to antimicrobial resistance or low antibiotic concentrations. These cells remain in the biofilm as persistent cells and are thought to contribute to chronic and recurrent infections [4]. Further clarification of the mechanism underlying persistent cell-mediated chronic infections may lead to a better understanding of how S. aureus causes chronic infections.

Although there are few epidemiological studies on extra-glandular involvement, arthritis due to Sjögren's syndrome is present in 16% of primary Sjögren's syndrome cases, with a high frequency of polyarthritis (71%) and monoarthritis (17%) [5]. The percentage of affected joints was 88%, with less than five joints affected, and the affected sites included 35% of proximal interphalangeal, 35% of metacarpophalangeal, 30% of main, 15% of the elbow, 10% of the knee, 10% of tarsal, 6% of the shoulder, 5% of metatarsophalangeal, and 3% of distal interphalangeal joints [5]. In addition, bone erosion on radiography was present in 5% and anti-CCP antibody positivity in 7% of patients, a low frequency in primary Sjögren's syndrome [5]. Thus, symmetric polyarthritis without radiographic bone erosion or anti-CCP antibodies is characteristic of joint lesions.

The patient's three affected joints, negative bone erosions, and anti-CCP antibodies were consistent with these characteristics; however, symmetry was not present. The patient's fingernail and Kevnellian skin findings, as well as the high frequency of complications between Sjögren's syndrome and rheumatoid arthritis (4%-31%), suggest that the patient may develop psoriatic arthritis or seronegative rheumatoid arthritis in the future [6]. However, since NSAIDs, colchicine, and PSL 10 mg were ineffective during treatment, arthritis caused by Sjogren's syndrome is more likely to occur.

Arthritis associated with an infection is considered to have an acute course. However, there may be subacute to chronic arthritis associated with S. aureus infection that is affected by the immunological factors of patients [7]. Older patients may not show the typical symptoms, and physical findings of various infectious diseases [8] and may not exhibit effective help-seeking behaviors [9]. General physicians, as system-specific specialists, should consider older patients’ conditions comprehensively during their clinical courses, such as joint pain, as in this case [10]. Patients with subacute-to-chronic refractory arthritis should be aggressively evaluated for infectious complications. Skin exfoliation is suspected to be a staphylococcal burn-like skin syndrome requiring immediate antibiotic intervention.

## Conclusions

In this case, a woman in her 60s had a subacute case of mono/minor arthritis with skin peeling, suspected to be a combination of arthritis caused by Sjögren's syndrome and S. aureus infection, which improved after treatment with PSL and antibiotics. However, S. aureus infections can be acute or chronic, involving persistent cells. Therefore, it is necessary to consider the possibility of bacterial co-infection in subacute cases of mono- and oligoarthritis with a course of approximately three weeks, as in this case.
